# Rapid and Efficient NO_2_ Sensing Performance of TeO_2_ Nanowires

**DOI:** 10.3390/s23229097

**Published:** 2023-11-10

**Authors:** Yunkun Shen, Kaili Wang, Hao Liu, Liping Chen, Zhihan Jin, Shancheng Yan

**Affiliations:** 1 College of Automation & College of Artificial Intelligence, Nanjing University of Posts and Telecommunications, Nanjing 210023, China; b2104313@njupt.edu.cn; 2 School of Integrated Circuit Science and Engineering, Nanjing University of Posts and Telecommunications, Nanjing 210023, China; 1222228419@njupt.edu.cn (K.W.); 2023221102@njupt.edu.cn (H.L.); 1223228118@njupt.edu.cn (Z.J.); 3 School of Geography and Biological Information, Nanjing University of Posts and Telecommunications, Nanjing 210023, China; 1023173009@njupt.edu.cn

**Keywords:** TeO_2_ nanowire, NO_2_ gas sensor, fast recovery time, length–diameter rate

## Abstract

Gas sensors play a pivotal role in environmental monitoring, with NO_2_ sensors standing out due to their exceptional selectivity and sensitivity. Yet, a prevalent challenge remains: the prolonged recovery time of many sensors, often spanning hundreds of seconds, compromises efficiency and undermines the precision of continuous detection. This paper introduces an efficient NO_2_ sensor using TeO_2_ nanowires, offering significantly reduced recovery times. The TeO_2_ nanowires, prepared through a straightforward thermal oxidation process, exhibit a unique yet smooth surface. The structural characterizations confirm the formation of pure-phase TeO_2_ after the anneal oxidation. TeO_2_ nanowires are extremely sensitive to NO_2_ gas, and the maximum response (defined as the ratio of resistance in the air to that under the target gas) to NO_2_ (10 ppm) is 1.559. In addition, TeO_2_ nanowire-based sensors can return to the initial state in about 6–7 s at 100 °C. The high sensitivity can be attributed to the length–diameter rate, which adsorbs more NO_2_ to facilitate the electron transfer. The fast recovery is due to the smooth surface without pores on TeO_2_ nanowires, which may release NO_2_ quickly after stopping the gas supply. The present approach for sensing TeO_2_ nanowires can be extended to other sensor systems as an efficient, accurate, and low-priced tactic to enhance sensor performance.

## 1. Introduction

With the worsening of environmental pollution, poisonous gas emissions, the energy crisis, and other problems, the demand for better selectivity, lower power consumption, and higher sensitivity of gas sensors is becoming increasingly urgent. However, toxic gas, like NO_2_, which can lead to an array of health complications such as nausea, respiratory tract irritation, asthma, asphyxiation, and, in severe cases, death [1], is hazardous. The recovery time of most NO_2_ sensors is about tens of or hundreds of seconds long, resulting in low efficiency and less accuracy in detection. As a result, a gas sensor nanomaterial needs to be exploited, which possesses a shorter sensing time.

Previously, various materials have been applied for sensing NO_2_; for example, Choi obtained that ZnO has gas sensitivity to NO_2_ gas, high selectivity, reasonable stability, and highly responsive sensing properties even in water vapor molecules. However, even if the ZnO is placed in a high-temperature environment, the fastest recovery time of it is still about 500 to 1000 s. The sample in this report was a 2D material with multiple rough holes. We believe this structure-stranded molecules’ movement is not conducive to releasing NO_2_ and would somewhat slow the recovery speed. Moreover, the selectivity of the gas sensor was reported to be poor [2].

In recent years, gas sensors based on metal oxide semiconductors have been applied mainly to this area because of their excellent performance, high stability, and floor price [3]. Materials with larger surface areas and smaller grain sizes are a priority for achieving high-performance gas sensors [4,5,6]. Therefore, quasi-one-dimensional oxide semiconductor nanomaterials are ideal for gas-sensitive applications due to their large surface-volume ratio and size effects. Mature gas-sensitive materials, such as SnO_2_ [7,8], ZnO [9], WO_3_ [10,11], Ga_2_O_3_ [12], and In_2_O_3_ [13] quasi-one-dimensional nanostructures, compared with relevant thin-film materials, have higher sensitivity, quicker response times, and more significant capability to detect low-concentration gases. However, they also have the disadvantages of poor selectivity, high sensing temperatures [14], and prolonged recovery time.

Previous studies of gas-sensing materials were usually according to n-type semiconductors for sensing investigations [15,16]. Nevertheless, few people pay attention to p-type semiconductors, like tellurium dioxide (TeO_2_), as gas sensors [17]. TeO_2_ is a widely used wide-band-gap semiconductor material. Because of its excellent acousto-optic and electro-optic properties [18], active optical devices widely make use of single-crystal TeO_2_ materials such as deflectors [19], modulators [20], tunable filters [21], hydrogen production [22,23], and potential antifungal agents [24]. When exposed to reducing gases, the outer layer of tellurium dioxide in semiconductor oxides reacts. Electrons trapped by oxygen are relayed to the semiconductor, decreasing the gas sensor’s resistance. Wansik Oum uses carbon-decorated TeO_2_, which has a more apparent corresponding coefficient at room temperature. A-C-decorated TeO_2_ nanowires have good selectivity to NO_2_ gas. The C-layer on the surface of the nanowires facilitates the transfer of holes. Nevertheless, the recovery time at room temperature is as long as 300 to 500 s. TeO_2_ in the report had a smooth surface and was covered by amorphous carbon (a-C) materials. This approach, however, hinders the release of NO_2_ molecules, resulting in slower recovery [25].

We believe that nanomaterials with smooth and undulating surfaces can accelerate the release rate of NO_2_ while expanding the effective surface area. At the same time, a rough or too-smooth surface can absorb more NO_2_ but will hinder its release rate, and the long recovery time may seriously impede the sensing application of nanowires. In this paper, TeO_2_ nanowires are oxidized via the natural oxidization of Te nanowires, which show high selectivity and sensitivity to NO_2_, and ultrafast recovery speed. Sensors based on TeO_2_ nanowires can revert to their initial state in approximately 6 to 7 s. The proposed gas sensor’s unique advantages include simple manufacturing, floor price, excellent selectivity, and good recovery time, which can be used in actual applications for NO_2_ sensing.

## 2. Experimental Details

### 2.1. Sample Preparation

**Figure 1:**
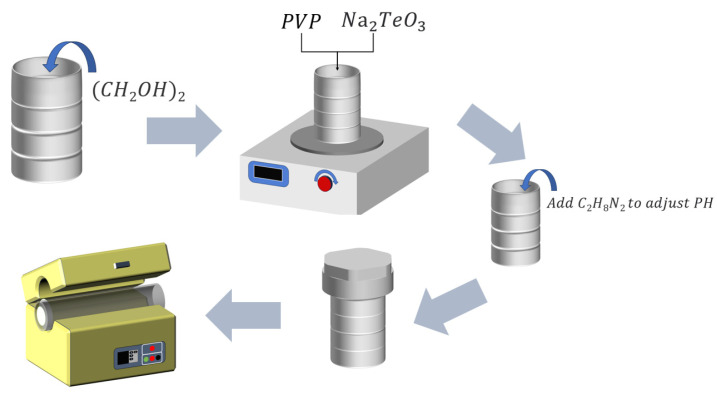
Scheme of the sample preparation process.

As shown in Figure 1, 25 mL of ethylene glycol solution was put into a polytetrafluoroethylene reaction kettle, followed by a 1.3 g polyvinylpyrrolidone (PVP) addition. The mixture was stirred with a magnetic stirring bar until it became transparent. Then, we added 1 mM of Na_2_TeO_3_ and stirred well for 2 h, followed by adding 1 mL of ethylenediamine solution to adjust the pH (the alkaline solution is convenient for replacing Te). The mixture was then placed in an oven at 180 °C for 3.5 h. After the reaction, it was repeatedly suction-filtered with water and ethanol. Then, it dried in a vacuum drying oven at 50 °C for 2 h to finally obtain a bulk product with a silvery-white and metallic luster. We put it into a 2 mL centrifuge tube, followed by adding 1 mL of ethanol, which was put in an ultrasonic machine for a short time and shaken several times to disperse the sample. Finally, it was dropped on a silicon wafer for observation.

To determine the best synthesis requirement for the oxidation of Te NWs to shape TeO_2_ nanowires, a series of heat treatments were performed at different temperature ranges (200 °C, 250 °C, 300 °C, and 350 °C) for a period of time. Ultimately, Te nanowires were annealed at the optimal temperature of 360 °C for 5.5 h and converted into high-quality nanowires using a high-temperature vacuum tube furnace [26].

### 2.2. Phase Analysis Method

The crystalline structure of the Te and TeO_2_ nanowires were characterized via X-ray diffraction (XRD, Bruker D8) using Cu Kα radiation (λ = 0.15406 nm) and further studied via high-resolution transmission electron microscopy (HRTEM JEOL-2100F, Tokyo, Japan). Observation of its morphology was conducted using field-emission-scanning electron microscopy (FE-SEM, JEOL JSM–7401F, Yangzhou, China). Raman spectra were obtained using a modified micro-Raman system (Invia Basic Renishaw, Nanjing, China) using an excitation laser wavelength of 514 nm, linking an Olympus microscope with a CCD detector and a Spex 1740 spectrometer. The transmission spectra were obtained using a UV-visible spectrophotometer (Thermo Scientific Evolution 201, Nanjing, China). A Thermo Scientific Nexsa XPS spectrometer with a monochromatic, micro-focused, low-power Al K-Alpha X-ray source selected photons with a 400 µm X-ray spot size and collected XPS spectra using a dual-focusing hemispherical analyzer with an energy of 50 eV.

### 2.3. Gas Analysis Method

The gas sensing experiments were conducted on an STP4 intelligent gas sensing analysis system (Nanjing Wisens Co., Ltd., China). To better estimate the performance of gas sensors, a method was adopted by measuring the resistance value of the gas sensors in a specified gas. The sensor’s resistance in the presence of air (Ra) and the target gas under a specified pressure (Rg) were measured, and the response was calculated using R = Ra/Rg. The recovery and response times calculation was based on the duration required to achieve a 90% resistance change when the target gas was introduced and halted separately.

## 3. Results and Discussion

### 3.1. Sample Characterization

**Figure 2:**
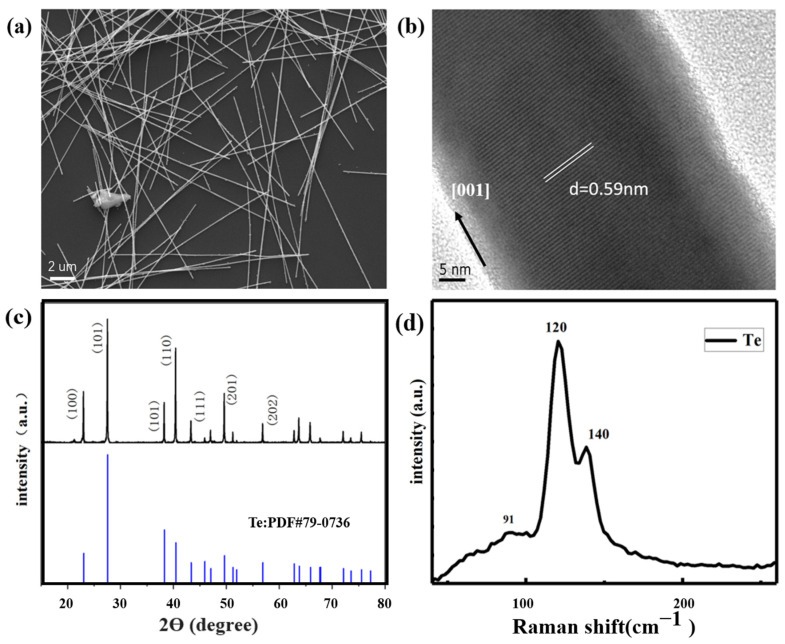
SEM and HRTEM analysis of Te nanowires. (**a**,**b**) XRD and Raman spectra of Te nanowires (**c**,**d**).

Figure 2a,b shows Te’s SEM and HRTEM images and the successfully synthesized Te lines of sufficient length. The SEM images are shown in Figure 2a. The solvothermal synthesis products were linear and had good morphology. The externally synthesized Te nanowires do not have apparent roughness on the outer surface; the surface is smooth. The internal structure is uniform without noticeable defects. This indicates that the synthesized Te nanowires possess high-quality characteristics. Nanowires were about 10- to 40-μm-long and about 60 nm in diameter. Figure 2b indicates a high-resolution transmission electron microscopy (HRTEM) image of tellurium nanowires. The interplanar spacing of the tellurium nanowires was 0.59 nm, indicating tellurium growth in the [001] direction.

The XRD pattern of the solvothermal synthesis product is shown in Figure 2c. No impurity peaks were found at the diffraction peak, indicating that our daily standard product tellurium was successfully synthesized via this method.

The Raman spectrum for the tellurium is shown in Figure 2d, with three distinct peaks at 91, 120, and 141 cm^−1^. The 91 cm^−1^ mode was caused by chain expansion, while the 120 and 141 cm^−1^ modes indicated that bond bending and bond stretching was induced, which is consistent with the conclusions of related papers [26].

**Figure 3:**
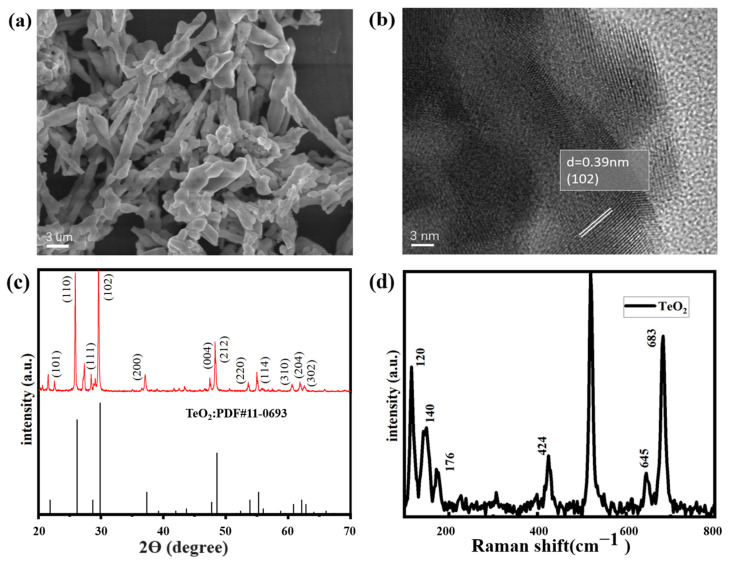
SEM and HRTEM analysis of TeO_2_ nanowires. (**a**,**b**) XRD and Raman spectra of TeO_2_ nanowires (**c**,**d**).

SEM and HRTEM images of the TeO_2_ are shown in Figure 3, indicating that a sufficiently long TeO_2_ image with a diameter of approximately 100–400 nm and a length of above 100–200 μm had been successfully synthesized. The shape was like a tree branch, the gas contact area was large, and the surface was not cracked.

Figure 3c shows the XRD pattern of the obtained product, indicating the formation of a high-quality tetragonal TeO_2_ crystal phase. All peaks are consistent with high-quality tetragonal TeO_2_ crystals, with lattice parameters (a = 4.796 A, c = 7.626 A, α = β = γ = 90°) which perfectly match the standard JCPDS data (JCPDS No.11-0693). The essence of highly crystalline TeO_2_ NWs is reflected in the appearance of sharp peaks. This means that the crystal is highly intact, with high purity and integrity, without significant defects or crystal distortions. This information is crucial for the study of the material’s crystal structure and quality.

Raman spectroscopy can be used to study the molecular vibrations and lattice vibrations of materials, providing information about material properties and phonon dynamics. The Raman spectra of pristine TeO_2_ nanowires, as shown in Figure 3d, indicated that the peak of 520 cm^−1^ was identified as the characteristic Raman peak of silicon, possibly originating from the Si substrate. The Raman distinct peak at 307 cm^−1^ matches the 2TA vibration mode of Si.

On the other side of the shield, the Raman peaks near 823 cm^−1^ and 229 cm^−1^ are related to the B1 vibration mode of TeO_2_, while the peaks near 427 cm^−1^ are related to the B2 mode. Additionally, the peak at 649 cm^−1^ is related to the A1 mode, and a relatively sharp peak was observed at 685 cm^−1^, corresponding to the E mode of TeO_2_ [27]. The sharp and distinct peaks observed in the Raman spectrum indicate that the TeO2 nanowires grown in the samples are of high quality.

**Figure 4:**
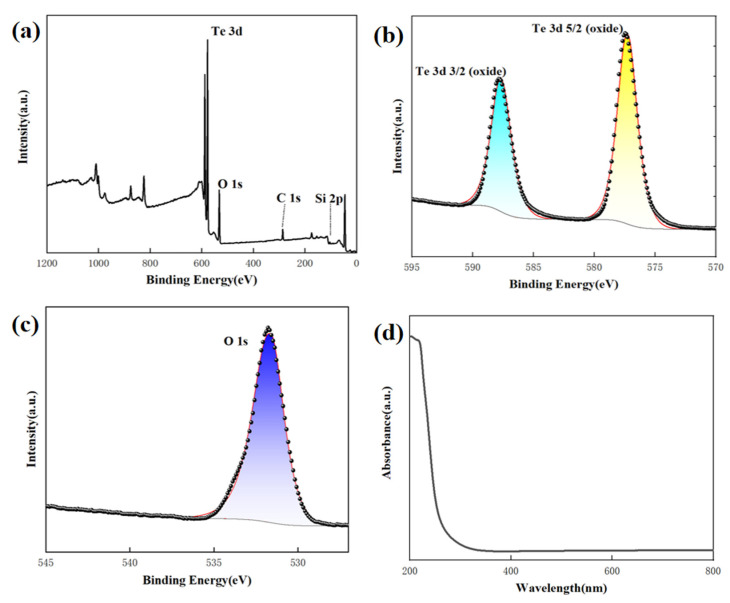
XPS spectra taken from the TeO_2_ nanowires. (**a**–**c**) and UV-vis absorption spectrum of TeO_2_ nanowires (**d**).

The analysis conducted using X-ray photoelectron spectroscopy (XPS) confirmed that the obtained product is TeO_2_. The XPS survey of the TeO_2_ nanowires in Figure 4a displays two prominent peaks corresponding to the Te and O elements. In Figure 4b, the Te 3d region reveals peaks at 587.88 and 577.38 eV for Te 3d_3/2_ and Te 3d_5/2,_ respectively, indicating the presence of TeO_2_ formation. Figure 4c displays the association of the peak at 530.6 eV in the O 1s binding energy region with TeO_2_. We conducted optical absorption spectroscopy tests to investigate the optical properties of the material. In Figure 4d, the optical absorption spectrum of the TeO_2_ nanowire samples is displayed within a wavelength range of 200–800 nm. Within this absorption spectrum, the maximum absorption peak is observed in the 200–400 nm wavelength range. This implies that TeO_2_ nanowire samples exhibit significant light absorption within this specific range of wavelengths. However, when the wavelength exceeds this range, the absorption intensity decreases sharply. This suggests that within the visible light and near-ultraviolet range, TeO_2_ nanowire samples exhibit pronounced light absorption, while the absorption significantly diminishes at longer wavelengths. This absorption spectrum characteristic is vital for understanding the material’s optical properties and potential applications.

### 3.2. Gas-Sensing Character

The resistance-based gas sensor uses the interactions between the gas and the resistance to detect the target gas. In this sensor, a resistors element is exposed to a gaseous environment. When the target gas is in contact with the resistive element, the target gas will chemically react or adsorb with the surface of the resistive element, resulting in a change in electrical resistance.

**Figure 5:**
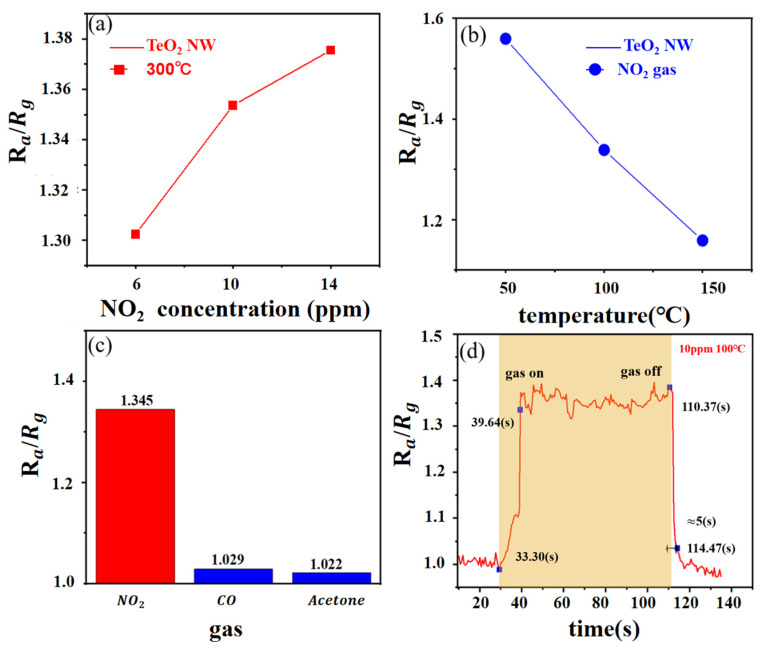
Gas-sensing character in different temperatures, different concentrations, and different gases.

Figure 5 displays the curve of the TeO_2_ gas sensor. This figure shows the transient resistance of the TeO_2_ nanowires to NO_2_ gas at 300 °C. It was clear that the p-type properties of the synthesized TeO_2_ nanowires have been demonstrated through their resistance changes. In Figure 5a, the responses of the TeO_2_ gas sensor to 6, 10, and 14 ppm of NO_2_ gas were 1.302, 1.354, and 1.376, separately. These results indicate that the response of the TeO_2_ gas sensor is highly significant at a given gas concentration. In the presence of the gas, the TeO_2_ nanowire resistance changes due to the interaction between the nitrogen dioxide and the nanowires. This interaction may be adsorption of gas molecules or chemical reaction with the surface of the nanowire, resulting in altered resistance. The response value of the sensor can be used to determine the presence and concentration of the target gas. As the concentration of nitrogen dioxide gas increases, the response value of the sensor also increases. This indicates that the TeO_2_ gas sensor has good sensitivity to nitrogen dioxide gas and enables efficient detection at lower gas concentrations

To evaluate the behavior of the TeO_2_ gas sensor at different temperatures, we conducted an in-depth study of gas sensing at multiple temperatures. Figure 5b shows the transient response and resistance plot of the TeO_2_ nanowire sensor to NO_2_ gas at 50, 100, and 150 °C, separately. The sensor responses were 1.559, 1.348, and 1.159, respectively. Therefore, it is concluded that the optimal experimental temperature of TeO_2_ for NO_2_ is 50 °C and that the corresponding effect gradually weakened with the temperature increase. At lower temperature, TeO_2_ nanowires have better nitrogen 2 adsorption capacity and higher sensitivity. When the temperature increases, the interaction of the target gas with the sensor surface may be weakened, thus weakening the response. This may be due to the increased temperature leading to a more intense movement of the gas molecules, which reduces the adsorption capacity or reaction rate of the gas and the sensor surface.

Figure 5c reveals the response and resistance value of the TeO_2_ nanowire sensor to interference gas at a temperature of 50 °C. The sensor responds to NO_2_, CO, and acetone at a concentration of 10 ppm 1.376, 1.029, and 1.022, respectively. TeO_2_ showed outstanding selectivity for NO_2_. The sensor showed clear selectivity against NO, with the highest response values to NO gas, much higher than the response values for CO and acetone. This suggests that the TeO_2_ nanowire sensor has higher selectivity and lower interference response to nitrogen 2.

Through the curve in Figure 5d, it can also be seen that the response and the recovery times were very short, which were about 6 s long. Table 1 compares the recovery time in this work to other NO_2_ gas-sensing materials. Furthermore, as shown in Table 1, we can observe faster recovery times for TeO_2_ nanowire sensors compared to other semiconducting gas-sensing materials. This means that the sensor is able to recover to the base state from the target gas. This is important for continuous monitoring and real-time detection because a faster recovery time allows the sensor to be ready for the next measurement or detection.

To explore the repeatability of the sensors, we built two sensors using the same materials and manufacturing procedures. The response of the two sensors to NO_2_ gas was almost the same, irrespective of the concentration in the range of 2–10 ppm, demonstrating its excellent repeatability.

## 4. Mechanism Analysis

The definite sensing framework of the TeO_2_ materials for NO_2_ gas still needs further research. A widely accepted explanation for the gas-sensing mechanism of gas-sensitive oxide is that when semiconductor oxides are exposed to reducing gases, tellurium dioxide outside the semiconductor responds to the reducing gas. The electrons are caught by oxygen and transferred to the semiconductor, thereby reducing the resistance of the gas sensor. As to n-type semiconductors, the resistance is reduced because of the enhancement of the concentration of electrons in the semiconductor. P-type semiconductors’ resistance rises when holes combine with electrons released by surface reactions [33]. When exposed to oxidative gas, the gas species serve as an acceptor, leading to a rise in the resistance of the n-type semiconductor and a reduction in the resistance of the p-type semiconductor. In this study, the sensor’s resistance decreasing under NO_2_ reveals that TeO_2_ nanowires exhibit p-type conductivity [34].

**Figure 6:**
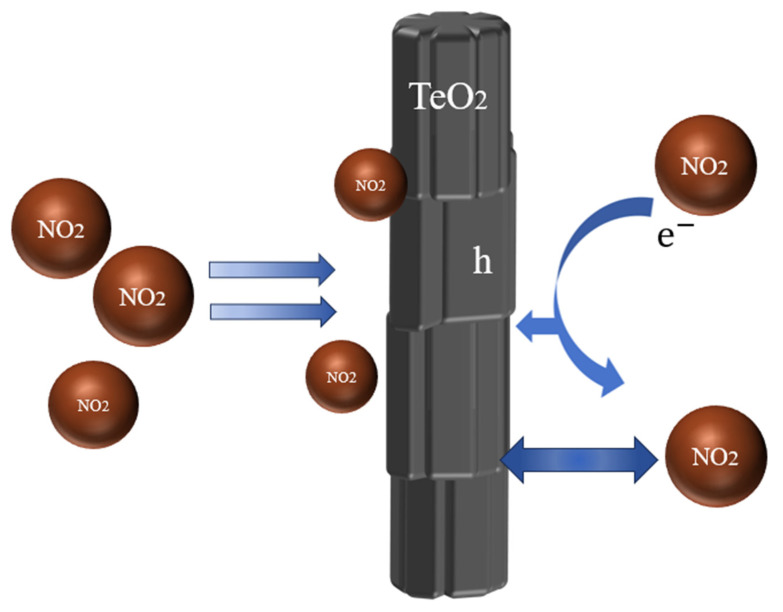
The mechanism of chemical interaction.

Upon exposure to the oxidizing gas NO_2_, the NO_2_ molecule is adsorbed on the surface of TeO_2_ as an acceptor. The NO_2_ molecule, with an unpaired electron response with the suspension bond on top of TeO_2_, successfully catches a solitary pair of electrons in the suspension bond and forms a free hole. The mechanism of chemical interaction between NO_2_ molecules and TeO_2_ nanowires (Figure 6) can describe the equation [33]:

$$TeO_{2}+NO_{2(g)}↔TeO_{2}-NO_{2\left(ad\right)}↔TeO_{2}^{+}-NO_{2\left(ad\right)}^{-}+h.$$

As a result, the hole concentration as the majority carrier in TeO_2_ nanowires rises, and the resistance diminishes. The sample presents a wrinkled morphology. The large surface area is the reason for the high sensitivity, as it potentially adsorbs more NO_2_, facilitating electron transfer. The quick recovery of the TeO_2_ nanowires results from their smooth, pore-less surface, which likely allows for the rapid release of NO_2_ once the gas supply is halted.

In conclusion, TeO_2_ nanowires have a tetragonal phase structure, are sensitive to NO_2_ gas, and have a typical p-type response. These results demonstrate the possibility of using TeO_2_ nanowires to manufacture low-power gas sensors.

## 5. Conclusions

In summary, TeO_2_ nanowire structures were prepared using a facile thermal oxidation method. The results showed that the TeO_2_ nanowires had a very fast reaction effect and recovery speed to NO_2_ gas. Their reaction time was about 10 s, while their recovery time was about 6 to 7 s at the temperature of 100 °C. At 50 °C, the response of TeO_2_ to NO_2_ reaches 1.559. The prepared TeO_2_ sensor realized low power consumption and high efficiency at a low temperature, showing applicability for safe and stable NO_2_ detection in the industrial field. The present work presents a valuable and potential design for developing NO_2_ gas sensors with excellent high-speed sensing performance.

## Figures and Tables

**Table 1:** Comparison of NO_2_ gas sensing properties of different sensors.

Sensing Materials	Gas	Concentration	Response	Response Time	Recovery Time	Reference
TeO_2_ nanowires	NO_2_	10 ppm	1.6	10 s	6 s	This work
Pr_2_Sn_2_O_7_/NiO	NO_2_	60 ppm	7.6	22 s	53 s	[28]
WO_3_ nanorods	NO_2_	50 ppm	2.02	96 s	81 s	[29]
CoFe_2_O_4_	NO_2_	100 ppm	110	15 s	18 s	[30]
a-C-decorated SnO_2_	NO_2_	10 ppm	13	800 s	3000 s	[31]
ZnO NPs-decorated CuO NWs	NO_2_	100 ppm	4	570 s	150 s	[32]

## Data Availability

Not available.
